# Unveiling the Impact of Cardiac Lymphatic Pathology on Diastolic Dysfunction and Therapeutic Potential of Lymphangiogenesis∗

**DOI:** 10.1016/j.jacbts.2023.07.010

**Published:** 2023-08-28

**Authors:** Maxim Itkin, Alexey Gurevich

**Affiliations:** Radiology Department, Penn Medicine, University of Pennsylvania, Philadelphia, Pennsylvania, USA

**Keywords:** cardiac diastolic dysfunction, cardiac lymphatic vessels, fibrosis, hypertrophy, inflammation

**Figure:**
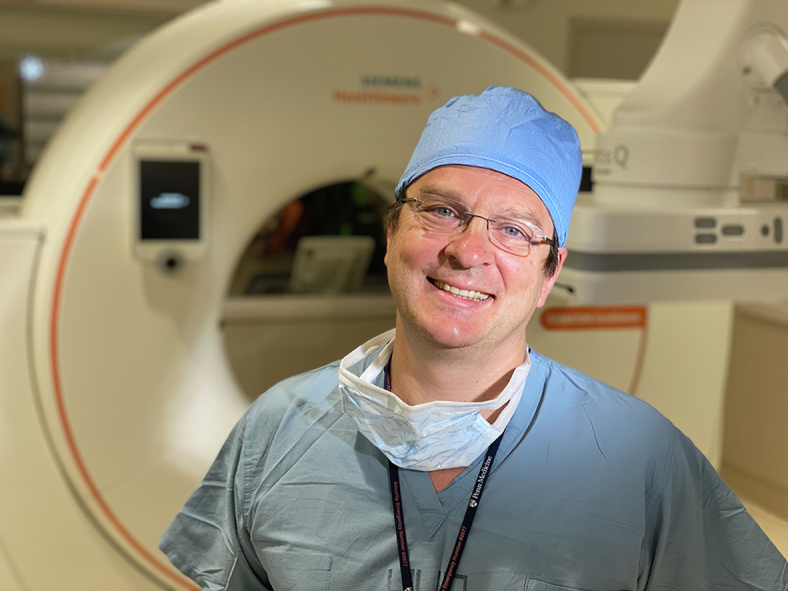

The lymphatic system is an integral part of the cardiovascular system and is responsible for removing fluid and proteins from the tissues. As such, lymphatic malfunction is part of the pathophysiological mechanism of tissue edema. However, because of complex anatomy, small size of the lymphatic vessels, and difficulty of introducing contrast, it has been overlooked over the arterial and venous circulations. Recently, the importance of the cardiac lymphatic system in myocardial pathophysiology was highlighted. Insufficient or disrupted lymphangiogenesis, both innate or acutely induced, plays a pathological role in myocardial remodeling and cardiac dysfunction. Poor cardiac lymphatic function then leads to myocardial edema, delayed resolution of inflammatory processes, and eventual myocardial interstitial fibrosis.^1^ To date, there have been limited therapies available to address this issue at its pathophysiological core. Whatever limited discoveries have been developed also suffered from the lack of appropriate animal models that provide adequate recapture of the disease and opportunity for intervention.

The study published in this issue of the *Journal of the American College of Cardiology* by Pu et al^2^ investigated the effects of cardiac lymphatic dysfunction on heart homeostasis under physiological and pathological conditions. A new mouse model of cardiac lymphatic vessel ablation was created to emulate this dysfunction. Several techniques, such as cardiac magnetic resonance imaging, fluorescein isothiocyanate–dextran experiments, echocardiography, histological analysis, real time quantitative polymerase chain reaction, and enzyme-linked immunosorbent assays, were used for the study. Furthermore, adipose-derived regenerative cells (ADRCs) were used to attempt to reconstruct the lymphatic network postablation 6 weeks after surgery. Last, a high-fat diet (HFD) was used to assess the impact of such a diet on heart disease progression when combined with lymphatic dysfunction.

Upon the induction of lymphatic dysfunction, marked by lymphatic vessel ablation, the study found an accumulation of lymph in the heart, reduced lymphatic drainage capacity, an increase in heart size and weight, and induced macrophage accumulation and cardiac fibrosis. These physiological alterations all point to significant distress on the cardiac system following lymphatic dysfunction. Notably, this distress was found to be further exacerbated in a synergistic inclusion of an HFD-induced obesity.

One key finding was that the lymphatic dysfunction had no impact on systolic function but significantly affected diastolic function. This is demonstrated by the increase in wall thickness and reduced E/A and E/e' values, both indicators of impaired diastolic function. The study suggests that the damage and alterations in the heart were likely caused by inflammation, given that the expression of inflammatory markers (TNF-α, IL1-β, and IL6), as well as profibrotic markers (COL1A1, TGF-β, and Postn) were all up-regulated. Cardiomyocyte hypertrophy markers were also up-regulated, favoring the pathophysiology to be that of an inflammatory fibrosis.

Finally, the diastolic dysfunction induced by ablated lymphatics was found to be alleviated by the implantation of ADRCs, suggesting that therapeutic lymphangiogenesis could potentially serve as an effective treatment for heart failure resulting from cardiac lymphatic dysfunction.

The establishment of a new mouse model to study cardiac lymphatic dysfunction paves the way for a deeper understanding of cardiovascular lymphatic pathophysiology. This is crucial because current knowledge of cardiac lymphatics and their role in heart disease is limited. This animal model can corroborate our current understanding and provide further valuable insights into the mechanisms of how lymphatic dysfunction contributes to various cardiac pathologies. The observed physiological alterations following lymphatic dysfunction shed light on the potential consequences of such a condition. The already mentioned results show that lymphatic dysfunction could lead to a chain of events including fluid accumulation, reduced drainage capacity, increased heart size and weight, and pathological changes such as macrophage accumulation and cardiac fibrosis.

Such observations are in line with the previously established findings that cardiac lymphatic dysfunction has significant contribution to myocardial interstitial fibrosis by delaying resolution of immune inflammatory responses, and increased secretions of profibrotic molecules from activated leukocytes that trigger myofibroblast differentiation in the myocardium. Additionally, the increased mechanical stress within the extracellular matrix caused by poor fluid outflow produces further differentiation of resident fibroblasts into profibrogenic myofibroblasts that produce more collagen and increase the stiffness of the myocardium.^3^

The study identified a clear relationship between aberrant cardiac lymphatics and diastolic dysfunction, which is significant because diastolic heart failure, particularly among the elderly, is challenging to treat and future research toward more targeted therapies for diastolic heart failure will be of much benefit. Notably, enhanced pathophysiological understanding can help clinicians better identify and diagnose heart issues with lymphatic etiologies.

Given that the deleterious effects of lymphatic dysfunction were exacerbated in the presence of HFD-induced obesity, these findings are particularly relevant in the context of the current obesity epidemic and high-fat Western diet. They suggest that therapies aimed at improving lymphatic function could also have benefits in treating obesity-related cardiac dysfunction, and that diet modification could have additional health benefits in these patient populations. However, translating this knowledge into clinical practice faces challenges, mainly because of the lack of established clinical methods for evaluating the cardiac lymphatic system.

One of the intriguing thoughts is the effect on heart function in patients post–heart transplant. Because the lymphatic channels are severed during surgery, there is an inherent obstruction of the cardiovascular lymphatic system in this patient population. In patients post–heart transplant who developed heart failure, the systolic function is often preserved, but left ventricular hypertrophy is present, similar to the findings in this study.^4^ Similarly, the radiofrequency catheter ablation for atrial fibrillation is known to cause left atrial diastolic dysfunction and pulmonary hypertension, the so called “stiff left atrium syndrome.”^5^ It is entirely possible that both of these dysfunctions are a result of the obliterated cardiovascular lymphatic vessels and therefore impaired lymphatic drainage from the myocardium.

The study further uncovers the potential of ADRCs in the treatment of cardiac conditions stemming from lymphatic dysfunction. The fact that ADRC implantation can promote lymphangiogenesis, suppress cardiac hypertrophy, reduce inflammation, and reverse fibrosis points toward their potential therapeutic use. This is especially important because induced tissue edema and chronic inflammatory fibrosis tend to further aggravate lymphatic dysfunction by directly obstructing lymphatic capillaries in the interstitial space.^6^ Inducing lymphangiogenesis could break this accumulative process and guide future research into regenerative medicine approaches for cardiac diseases.

Although the study shows promising results, these discoveries are based on a mouse model, emphasizing the need for further research. This methodology can become a stepping stone for future translational projects that will aim to bring these findings into a clinical setting, both to confirm these outcomes in humans and to explore potential therapeutic applications of ADRCs in treating heart failure caused by lymphatic dysfunction.

In conclusion, Pu et al^2^ highlight the overlooked significance of heart lymphatics in the global picture of cardiovascular disease. Novel methods of studying the involvement of lymphatics in myocardial pathophysiology can help us deepen the known landscape of heart disease and pave the way for new translational approaches to treating diastolic heart failure with lymphatic involvement.

## Funding Support and Author Disclosures

The authors have reported that they have no relationships relevant to the contents of this paper to disclose.
